# Pharmacological Targeting of Sphingosine Kinases Impedes HIV-1 Infection of CD4 T Cells through SAMHD1 Modulation

**DOI:** 10.1128/jvi.00096-22

**Published:** 2022-04-12

**Authors:** Rachel S. Resop, Alberto Bosque

**Affiliations:** a Department of Microbiology, Immunology and Tropical Medicine, The George Washington University, Washington, DC, USA; Icahn School of Medicine at Mount Sinai

**Keywords:** CD4, HIV-1, SAMHD1, sphingosine-1-phosphate, sphingosine kinases

## Abstract

Sphingosine-1-phosphate (S1P) is a sphingolipid modulator of a myriad of cellular processes, and therapeutic targeting of S1P signaling is utilized clinically to treat multiple sclerosis. We have previously shown that functional antagonism of S1P receptors reduces cell-free, cell-to-cell, and latent HIV-1 infection in primary CD4 T cells. In this work, we examined whether targeting sphingosine kinase 1 or 2 (SPHK1/2) to inhibit S1P production would prevent infection using multiple HIV-1 primary isolates and infectious molecular clones. SPHK inhibition reduced HIV transmission between primary CD4 T cells in both cell-to-cell transmission and pretreatment coculture models. Mechanistically, pharmacological inhibition of SPHK reduced susceptibility to infection primarily by downregulating phosphorylated SAMHD1 (pSAMHD1), enhancing the activity of this innate HIV-1 restriction factor. Furthermore, genetic disruption of either SPHK1 or SPHK2 by CRISPR/Cas9 reduced phosphorylation of SAMHD1, demonstrating the role of these kinases in modulation of SAMHD1 activity. The effect of SPHK inhibition on limiting HIV-1 infection in CD4 T cells was observed irrespective of the biological sex or age of the donor, with neither variable significantly influencing the effectiveness of SPHK inhibition. Our results demonstrate that targeting SPHK inhibits transmission of HIV-1 via modulation of SAMHD1 phosphorylation to decrease permissiveness to infection in CD4 T cells and suggests that therapeutic targeting of this pathway early in infection enables development of strategies to prevent establishment of infection and hinder cell-to-cell transmission of HIV-1.

**IMPORTANCE** HIV-1 infection, once established, requires lifelong treatment due to the ability of the virus to maintain latent infection in its host and become reactivated during an interruption in antiretroviral treatment (ART). Although preventing transmission and acquisition of HIV is an important goal, no ART thus far have exploited harnessing a component of the host immune system to combat transmission of the virus. We have previously shown that inhibition of sphingosine-1-phosphate (S1P) receptors, a component of S1P signaling, reduces HIV-1 infection in human CD4 T cells. We therefore investigated inhibition of sphingosine kinases, another element of this signaling system, in this work. We found that inhibition of sphingosine kinases 1 and 2 (SPHK1/2) could reduce HIV-1 transmission, both among CD4 T cells and between macrophages and CD4 T cells. Our research therefore suggests that therapeutic targeting of SPHK or S1P receptors may aid in the development of strategies to prevent establishment and transmission of HIV-1 infection among immune cells.

## INTRODUCTION

Human immunodeficiency virus (HIV-1) has been a global health burden for over 30 years, with nearly 40 million people currently living with the virus worldwide. Due to the ability of HIV-1 to establish latent infection by integration of its genome into that of the host, permitting viral transcription following future activation of resting cells, treatment is lifelong for individuals living with HIV (1–8). Currently available antiretroviral therapies (ART) control viral load by targeting various stages of the viral life cycle, but many individuals experience off-target effects (9), and no antiretrovirals have focused on immunomodulatory compounds directed toward a component of the immune system. Such a strategy could have efficacy in a wide range of individuals while avoiding some of the side effects observed with ART. Therefore, exploration of alternative and novel methods to harness host cellular machinery to combat HIV-1 are needed.

Sphingosine-1-phosphate (S1P) is a sphingolipid modulator of a myriad of cellular processes, and its therapeutic targeting is under clinical investigation. The effects of S1P are mediated through its receptors, sphingosine-1-phosphate receptors 1 to 5 (S1PR1-5; reviewed in reference 10). We and others have shown that S1P receptor 1 (S1PR1) and S1P receptor 4 (S1PR4) are expressed on various subsets of immune cells, including CD4 T cells, and can be modulated by a repertoire of agonists and specific antagonists (11–14). Sphingosine kinases (SPHKs), of which two isoenzymes exist, SPHK1 and SPHK2, are also present within multiple immune cell subsets, including CD4 T cells, and are responsible for the generation of S1P via phosphorylation of sphingosine (15–17). S1P is a player in inflammation and various disease states (10, 18, 19), and S1P modulators have been thoroughly investigated as potential cancer therapeutics (20–22). However, despite its crucial role as an immune signaling mainstay, there is a paucity of information on the interplay between S1P signaling and HIV-1 pathogenesis.

We have recently demonstrated that inhibition of S1P receptors with the functional antagonist FTY720 (fingolimod/gilenya) inhibits HIV-1 infection of primary CD4 T cells by multiple mechanisms. Inhibition of infection with FTY720 involves blocks at multiple levels of the HIV-1 life cycle and suggests the requirement of S1P binding to S1P receptors for the progression of infection in CD4 T cells (14). We therefore next sought to determine the role of availability of S1P itself in cell-to-cell transmission of HIV-1 by inhibiting SPHKs, reasoning that hindering the conversion of sphingosine to S1P may phenocopy the effect of FTY720. Although we have recently shed light on the involvement of S1PR in HIV-1 infection in CD4 T cells, to our knowledge this is the first investigation into the role of SPHKs in HIV-1 infection.

FTY720 is clinically approved for treatment of multiple sclerosis (MS) due to its ability to sequester lymphocytes and reduce the autoimmune reaction (23, 24) and is well tolerated when taken daily (25, 26). Harnessing the established safety and efficacy of FTY720, we built upon our recent report of its ability to reduce HIV-1 cell-to-cell transmission (14) and hypothesized that reduced availability of S1P via SPHK inhibition also may be effective at inhibiting HIV-1 infection and, importantly, specific, as one or both isoforms of sphingosine kinase can be targeted. Using primary cell models of HIV-1 cell-to-cell transmission in CD4 T cells (27) and a panel of HIV-1 primary isolates and molecular clones, we examined whether targeting SPHK1 or SPHK2 with specific or dual antagonists either during pretreatment or cell-to-cell transmission of HIV-1 would alter transmission dynamics. In this work, we report that inhibition of sphingosine kinases reduces the susceptibility of CD4 T cells to infection primarily via modulation of SAMHD1 phosphorylation. Genetic disruption of SPHK1 or SPHK2 demonstrates the role of these kinases in SAMHD1 phosphorylation. We therefore further advance the body of knowledge of the role of S1P signaling in HIV transmission in primary CD4 T cells and suggest specific and novel potential HIV-1 therapeutic candidates.

## RESULTS

### Sphingosine kinase or S1P receptor inhibition reduce HIV-1 infection in primary CD4 T cells.

**FIG 1:**
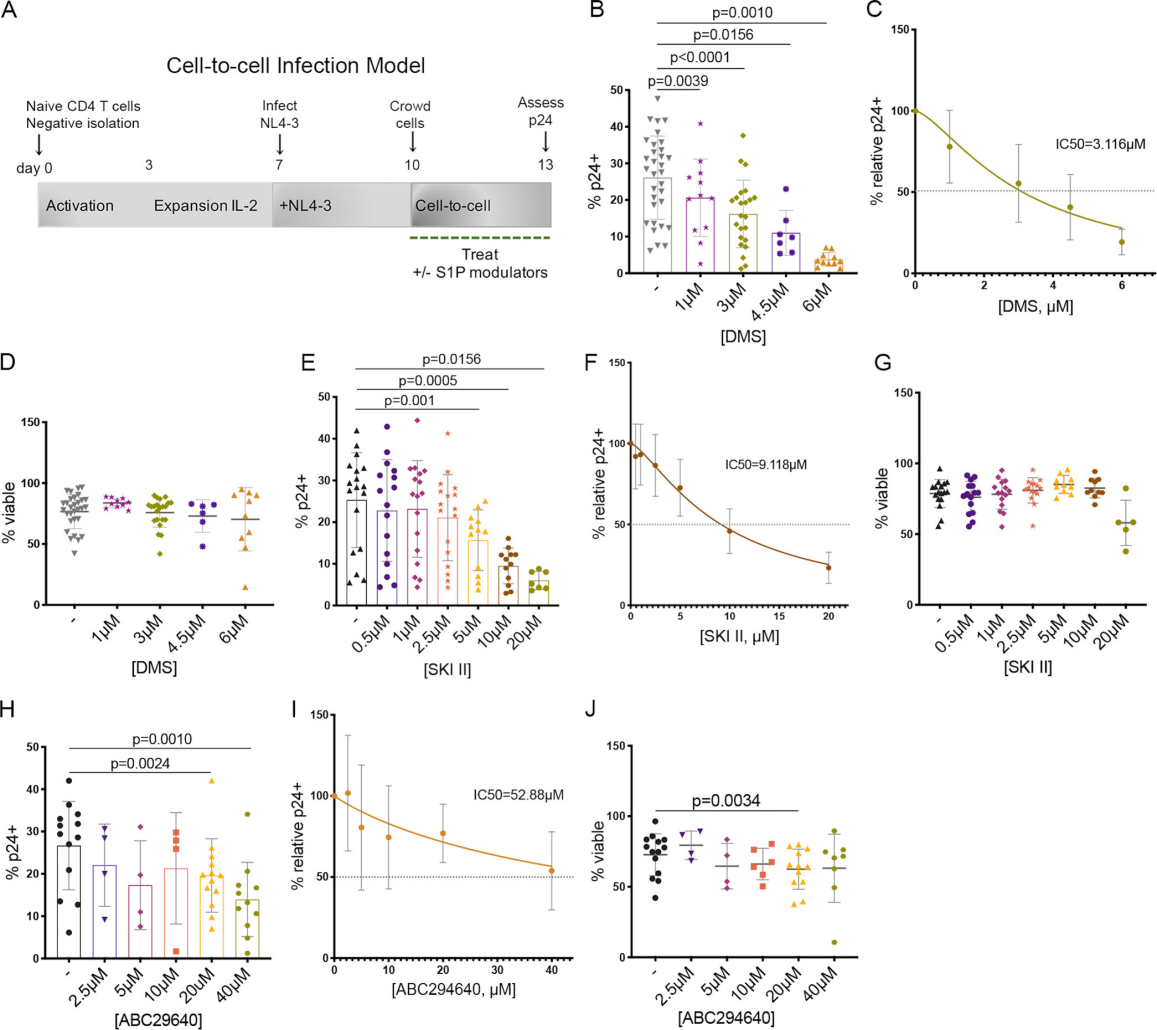
SPHK inhibition reduces HIV-1 cell-to-cell transmission in primary CD4 T cells. (A) Primary T_CM_ model of HIV infection indicating infection at day 7 followed by crowding with FTY720, DMS, SKI II, or ABC294640 treatment days 10 to 13 and assessment of HIV-1 p24-Gag by flow cytometry. (B) Summary of 72-h DMS treatment (1 to 6 μM) during crowding of NL4-3-infected CD4 T cells. Data from at least 7 individual donors with means ± standard deviations (SD) are shown (Wilcoxon matched-pair signed-rank tests). (C) Dose-response curve for DMS during NL4-3 cell-to-cell transmission expressed as a relative percentage of infected cells (p24^+^) versus concentration of DMS (IC_50 _= 3.116 μM), means ± SD are shown. (D) Summary of viability of NL4-3 infected CD4 T cells treated for 72 h (days 10 to 13) with 1 to 6 μM DMS, *n* = at least 6 donors per condition, means ± SD are shown (Wilcoxon matched-pair signed-rank tests). (E) Summary of 72-h SKI II treatment (0.5 to 20 μM) during crowding of NL4-3-infected CD4 T cells. Data from at least 7 individual donors with means ± SD are shown (Wilcoxon matched-pair signed-rank tests). (F) Dose-response curve for SKI II during NL4-3 cell-to-cell transmission expressed as the relative percentage of infected cells (p24^+^) versus concentration of SKI II (IC_50_ = 9.118 μM), means ± SD are shown. (G) Summary of viability of NL4-3 infected CD4 T cells treated for 72 h (days 10 to 13) with 0.5 to 20 μM SKI II, *n* = at least 5 donors per condition, means ± SD are shown (Wilcoxon matched-pair signed-rank tests for all comparisons). (H) Summary of 72-h ABC294640 treatment (2.5 to 40 μM) during crowding of NL4-3-infected CD4 T cells. Data from at least 4 individual donors with mean ± SD is shown (Wilcoxon matched-pair signed-rank tests). (I) Dose-response curve for ABC294640 during NL4-3 cell-to-cell transmission expressed as the relative percentage of infected cells (p24^+^) versus concentration of ABC294640 (IC_50_ = 52.88 μM), means ± SD are shown. (J) Summary of viability of NL4-3-infected CD4 T cells treated for 72 h (day 10 to 13) with 2.5 to 40 μM ABC294640, *n* = at least 4 donors per condition, means ± SD are shown (Wilcoxon matched-pair signed-rank tests for all comparisons).

We have recently demonstrated that treatment with the S1PR functional antagonist FTY720/fingolimod inhibits cell-free and cell-to-cell transmission of HIV-1 in a dose-dependent manner in primary CD4 T cells (14). To examine the requirement of SPHK for HIV-1 progression, we utilized a dual inhibitor of both kinases, *N*,*N*-dimethyl sphingosine (DMS), and specific inhibitors of SPHK1 (SKI II) and SPHK2 (ABC294640). Using a primary cell model of HIV infection, activated CD4 T cells were infected with the CXCR4 (X4)-tropic HIV-1 molecular clone NL4-3 at day 7 and treated with 1 to 6 μM DMS, 0.5 to 20 μM SKI II, 2.5 to 40 μM ABC294640, or 100 nM FTY720, which we utilized as a positive control based on our previous studies (14), for 72 h while crowding cells in 96-well plates to promote HIV-1 cell-to-cell transmission (Fig. 1A). Following 3 days of crowding, HIV p24-Gag was assessed by flow cytometry. We observed that dual SPHK inhibition with DMS resulted in significant inhibition of HIV-1 cell-to-cell transmission (DMS 50% inhibitory concentration [IC_50_] = 3.116 μM; *P* = 0.0039 for 1 μM, *P* < 0.0001 for 3 μM, *P* = 0.0156 for 4.5 μM, and *P* = 0.0010 for 6 μM) (Fig. 1B and C). Viability was not significantly affected relative to HIV-infected untreated cells by DMS, yet the uppermost concentration (6 μM) appeared to reduce viability in some donors; we therefore focused on lower concentrations in subsequent experiments (Fig. 1D). Specific inhibition of SPHK1 with SKI II also resulted in inhibition of infection (SKI II IC_50_ = 9.118 μM; *P* = 0.001 for 5 μM, *P* = 0.0005 for 10 μM, and *P* = 0.0156 for 20 μM) without a loss of viability at concentrations up to 10 μM (Fig. 1E to G). Inhibition of SPHK2 with ABC294640 moderately reduced p24-Gag expression but also impacted viability at the uppermost concentrations tested (Fig. 1H to J). As SPHK inhibition, in addition to S1PR modulation with FTY720, hinders HIV-1 cell-to-cell transmission, our results suggest that SPHK1 and SPHK2 are involved in HIV-1 infection of primary CD4 T cells and represent unique targets toward halting HIV-1 infection.

### Transmission of HIV-1 primary isolates and molecular clones is reduced by inhibition of sphingosine kinases.

**FIG 2:**
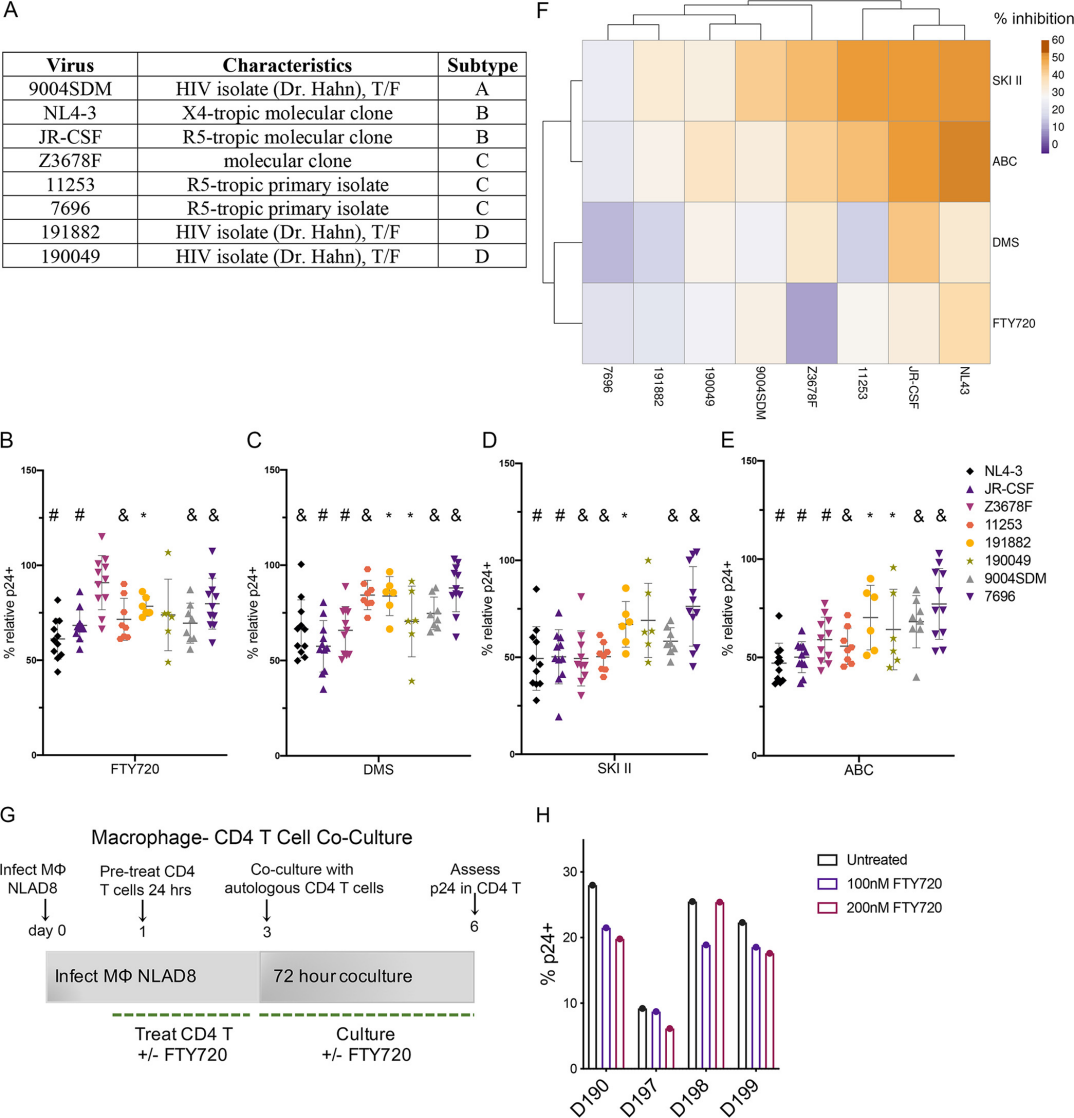
Inhibition of HIV-1 primary isolates and molecular clones with SPHK/S1PR modulation. (A) HIV-1 primary isolates and molecular clones utilized in cell-to-cell infection experiments; T/F, transmitted/founder virus. (B) Summary of 72-h FTY720 treatment (100 nM) during crowding of CD4 T cells infected with a panel of HIV-1 primary isolates/molecular clones. Data from 6 to 11 individual donors with means ± SD are shown (Wilcoxon matched-pair signed-rank tests). (C) Summary of 72-h DMS treatment (3 μM) during crowding of CD4 T cells infected with a panel of HIV-1 primary isolates/molecular clones. Data from 6 to 11 individual donors with means ± SD are shown (Wilcoxon matched-pair signed-rank tests). (D) Summary of 72-h SKI II treatment (10 μM) during crowding of CD4 T cells infected with a panel of HIV-1 primary isolates/molecular clones. Data from 6 to 11 individual donors with means ± SD are shown (Wilcoxon matched-pair signed-rank tests). (E) Summary of 72-h ABC294640 treatment (20 μM) during crowding of CD4 T cells infected with a panel of HIV-1 primary isolates/molecular clones. Data from 6 to 11 individual donors with means ± SD are shown (Wilcoxon matched-pair signed-rank tests). For panel B to E, the percent p24 relative to solvent control (ethanol [EtOH] for FTY720/DMS and dimethyl sulfoxide [DMSO] for SKI II and ABC294640) is shown; *, *P* ≤ 0.05; &, *P* ≤ 0.01; #, *P* ≤ 0.001. (F) Cluster analysis of percent inhibition attributed to treatment with FTY720, DMS, SKI II, and ABC294640 during cell-to-cell transmission with all HIV-1 primary isolates/molecular clones. (G) Schematic of infection of macrophages and coculture with autologous CD4 T cells with pre- and posttreatment (24 and 72 h, respectively) with 100 to 200 nM FTY720. (H) Summary of macrophage-CD4 T cell coculture experiments, *n* = 4; nomenclature refers to the identification number of each donor.

We next examined whether sphingosine kinase modulation would effectively inhibit HIV-1 transmission in a panel of HIV-1 primary isolates and infectious molecular clones representing a range of HIV-1 subtypes, including a subtype A primary isolate (9004SDM), two subtype B molecular clones (NL4-3 and JR-CSF), three subtype C isolates/molecular clones (Z3678F, 11253, and 7697), and two subtype D primary isolates (191882 and 190049 [28]) (Fig. 2A). Primary CD4 T cells were infected as previously described (Fig. 1A) and treated with SPHK inhibitors in concentrations approximating the IC_50_s calculated for Fig. 1: 3 μM DMS and 10 μM SKI II, as well as 20 μM ABC294640, or our positive control, FTY720 (100 nM, as previously used [14]), from day 10 to 13. As can be seen in Fig. 2B to E, treatment with both FTY720 and SPHK modulators reduced cell-to-cell transmission in the majority of primary HIV-1 isolates/molecular clones examined, and the extent to which inhibition occurred varied by virus and inhibitor, with some viruses more resistant to reduced transmission mediated by pharmacological inhibition of SPHK. We performed cluster analysis of the percent inhibition of HIV-Gag p24 for each inhibitor and virus (Fig. 2F), which demonstrated that the SPHK1 and SPHK2 inhibitors SKI II and ABC294640 have greater inhibitory activity relative to DMS and FTY720 in the majority of primary isolates/molecular clones tested.

The early events following initial HIV-1 transmission involve the transfer of HIV virions from infected macrophages to CD4 T lymphocytes (29). As an extension of our transmission studies, we were therefore interested in determining whether S1P modulation would affect transmission of virus between macrophages and CD4 T cells. Macrophages were derived from peripheral blood mononuclear cells (PBMCs) in the presence of 100 ng/mL macrophage colony-stimulating factor (M-CSF) and 10% sodium pyruvate (modification of references 30 and 31) and then infected with the R5-tropic HIV molecular clone NL-AD8 for 72 h. Three days later, we cocultured autologous CD4 T cells pretreated for 24 h with 100 to 200 nM FTY720 with NL-AD8-infected macrophages for 72 h (maintaining FTY720 in culture) and enumerated the percentage of p24^+^ CD4 T cells (Fig. 2G). We observed that treatment of CD4 T cells with FTY720 also inhibited transinfection from macrophages to CD4 T cells, indicating the potential of targeting S1P signaling to reduce transfer of HIV virions in initial transmission events (Fig. 2H).

### Target cell susceptibility to infection by a panel of HIV-1 primary isolates and molecular clones is reduced by SPHK modulation.

**FIG 3:**
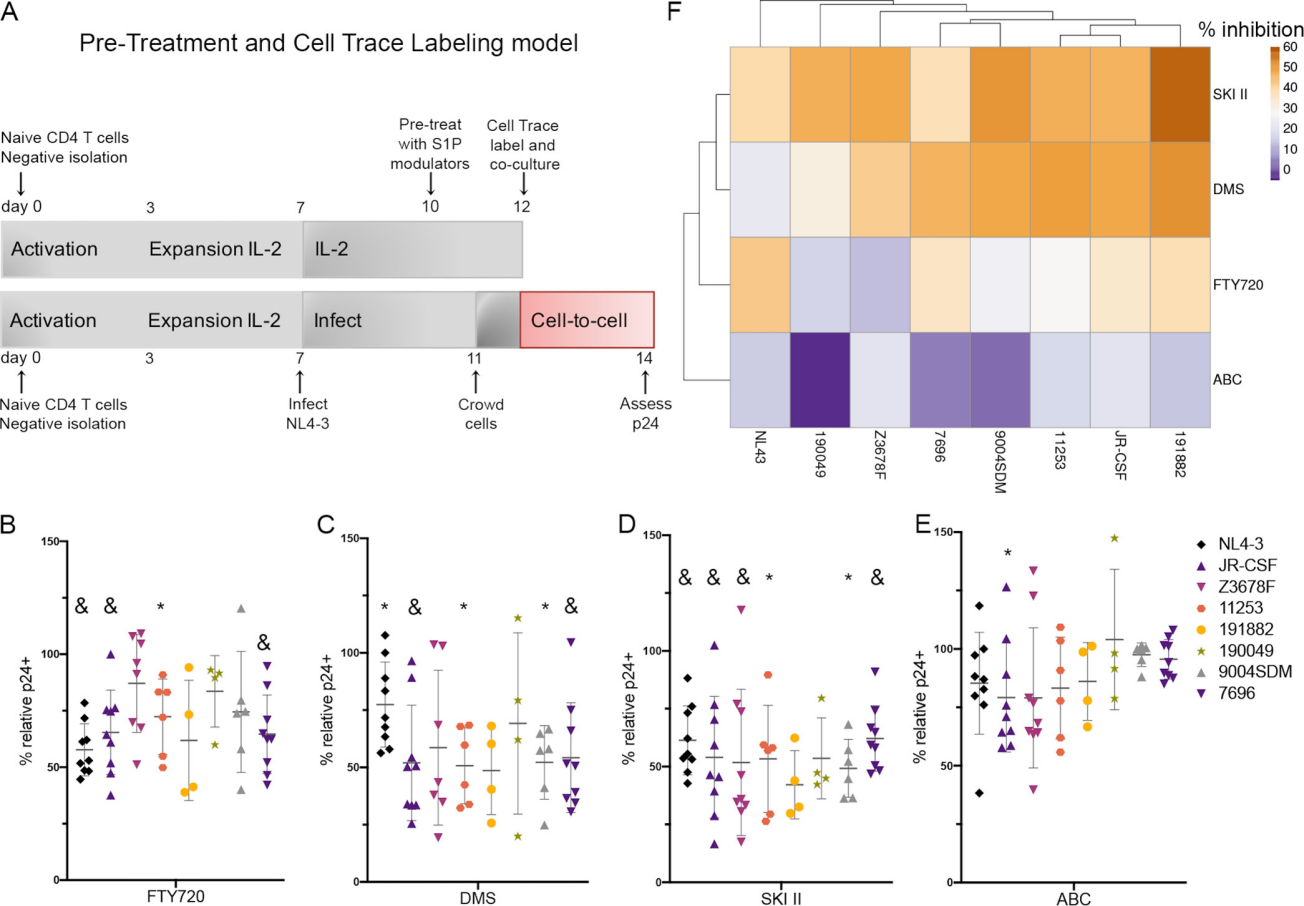
Inhibition of infection of target cells by HIV-1 primary isolates and molecular clones with SPHK/S1PR modulation. (A) Schematic of pretreatment and cell trace labeling model. (B) Summary of 48-h FTY720 pretreatment (100 nM) followed by coculture with CD4 T cells infected with a panel of HIV-1 primary isolates/molecular clones. Data from 4 to 9 individual donors with means ± SD are shown (Wilcoxon matched-pair signed-rank tests). (C) Summary of 48-h DMS pretreatment (3 μM) followed by coculture with CD4 T cells infected with a panel of HIV-1 primary isolates/molecular clones. Data from 4 to 9 individual donors with means ± SD are shown (Wilcoxon matched-pair signed-rank tests). (D) Summary of 48-h SKI II pretreatment (10 μM) followed by coculture with CD4 T cells infected with a panel of HIV-1 primary isolates/molecular clones. Data from 4 to 9 individual donors with means ± SD are shown (Wilcoxon matched-pair signed-rank tests). (E) Summary of 48-h ABC294640 pretreatment (20 μM) followed by coculture with CD4 T cells infected with a panel of HIV-1 primary isolates/molecular clones. Data are from 4 to 9 individual donors with means ± SD are shown (Wilcoxon matched-pair signed-rank tests). For panels B to E, the % p24 relative to solvent control (EtOH for FTY720/DMS and DMSO for SKI II and ABC294640) is shown; *, *P* ≤ 0.05; &, *P* ≤ 0.01; #, *P* ≤ 0.001. (F) Cluster analysis of percent inhibition attributed to pretreatment with FTY720, DMS, SKI II, and ABC294640 with all HIV-1 primary isolates/molecular clones.

Our previous studies of FTY720 in primary CD4 T cells demonstrated that the inhibition of infection due to S1P receptor modulation was likely primarily an effect specific to target cells, rendering them less susceptible to incoming virus infection (14). Therefore, we hypothesized that the inhibitory activity of SPHK modulators was also the result of reduced infectivity of incoming virions in target cells. To examine this, we developed a coculture infection model in which target cells were pretreated for 48 h with 100 nM FTY720, 3 μM DMS, 10 μM SKI II, or 20 μM ABC294640 (days 10 to 12) and then labeled with Cell Trace Yellow in fresh medium with treatment removed and cocultured with primary CD4 cells infected with a panel of HIV-1 primary isolates and molecular clones representing a range of HIV-1 subtypes as described above (coculture model, Fig. 3A; viruses, Fig. 2A). Following 2 days of coculture and crowding, the percent p24^+^ cells in the labeled, pretreated (or untreated) fraction was determined. As can be seen from Fig. 3B to E, pretreatment with SPHK inhibitors or FTY720 resulted in decreased transmission of the majority of HIV-1 primary isolates/molecular clones to target cells, and the extent to which this occurred varied by virus and treatment. Cluster analysis revealed an intriguing difference in the effect of SPHK modulators during pretreatment (Fig. 3F) versus cell-to-cell transmission (Fig. 2F), with the SPHK1 inhibitor SKI II and the dual inhibitor DMS now clustering together, indicating the greatest activity against most viruses in this model system. ABC294640, which in cell-to-cell transmission studies effectively reduced HIV-1 p24 across the majority of isolates and molecular clones, was the least effective at reducing infection during pretreatment, which suggests a different mechanism of action for this inhibitor.

### Effect of biological sex on SPHK inhibition and HIV-1 infection.

**FIG 4:**
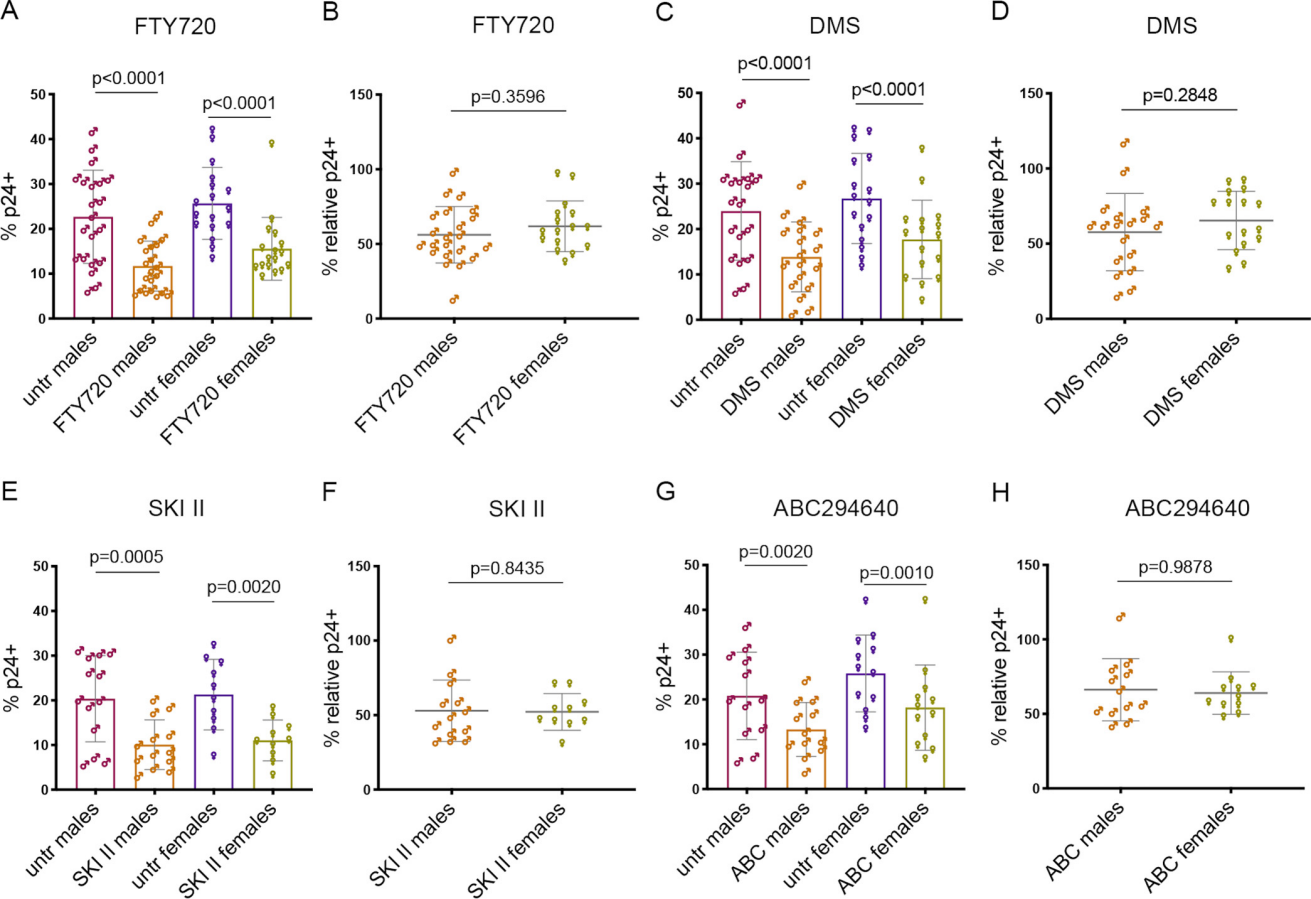
Effect of biological sex on sphingosine kinase inhibition. (A) Summary of 72-h 100 nM FTY720 treatment during cell-to-cell transmission in male versus female donors, *n* = 22 male and *n* = 16 female donors (Wilcoxon matched-pair signed-rank tests). (B) Summary of the percent relative p24^+^ CD4 T cells in male versus female donors treated with 100 nM FTY720 for 72 h during crowding, *n* = 22 male and *n* = 16 female donors (Mann-Whitney unmatched pairs two-tailed *t* test). (C) Summary of 72-h 3 μM DMS treatment during cell-to-cell transmission in male versus female donors, *n* = 18 male and *n* = 16 female donors (Wilcoxon matched-pair signed-rank tests). (D) Summary of the percent relative p24^+^ CD4 T cells in male versus female donors treated with 3 μM DMS for 72 h during crowding, *n* = 18 male and *n* = 16 female donors (Mann-Whitney unmatched pairs two-tailed *t* test). (E) Summary of 72-h 10 μM SKI II treatment during cell-to-cell transmission in male versus female donors, *n* = 13 male and *n* = 10 female donors (Wilcoxon matched-pair signed-rank tests). (F) Summary of the percent relative p24^+^ CD4 T cells in male versus female donors treated with 10 μM SKI II for 72 h during crowding, *n* = 13 male and *n* = 10 female donors (Mann-Whitney unmatched pairs two-tailed *t* test). (G) Summary of 72-h 20 μM ABC294640 treatment during cell-to-cell transmission in male versus female donors, *n* = 12 male and *n* = 11 female donors (Wilcoxon matched-pair signed-rank tests). (H) Summary of the percent relative p24^+^ CD4 T cells in male versus female donors treated with 20 μM ABC294640 for 72 h during crowding, *n* = 12 male and *n* = 11 female donors (Mann-Whitney unmatched pairs two-tailed *t* test). For all panels, means ± standard deviation are shown.

There is yet much to be learned regarding the effectiveness of antiviral agents across biological sexes. We therefore next questioned whether reduction of productive infection following SPHK inhibition would be observed equally among CD4 T cells expanded from biologically male and female blood donors. We generated CD4 T cells and infected them with NL4-3 as in Fig. 1A from an approximately equal number of male and female donors and treated them with 3 μM DMS, 10 μM SKI II, and 20 μM ABC294640, or our positive control, FTY720 (100 nM, as previously used [14]), for 72 h while crowding cells as described above. We then determined the relative inhibition of infection in CD4 T cells expanded from both sexes. Inhibition of infection with FTY720 resulted in similar effects across sexes (43.86% inhibition in males, standard deviation, 18.94; 38.19% inhibition in females, standard deviation, 16.94; Fig. 4A and B), as did DMS (42.33% inhibition in males, standard deviation 25.77; 34.62% inhibition in females, standard deviation 19.43; Fig. 4C and D) and SKI II (47.00% inhibition in males, standard deviation 20.62; 47.8% inhibition in females, standard deviation 12.33; Fig. 4E and F). ABC294640 also promoted a reduction in productive infection in both biological sexes (33.75% inhibition in males, standard deviation 20.87; 36.0% inhibition in females, standard deviation 14.27), yet in some donors this was observed concomitantly with reduced viability, suggesting that SPHK2 inhibition in the 20 to 40 μM range either promoted death of infected cells or reduced viability overall (Fig. 4G and H).

### SPHK inhibition of HIV infection occurs irrespective of donor age.

**FIG 5:**
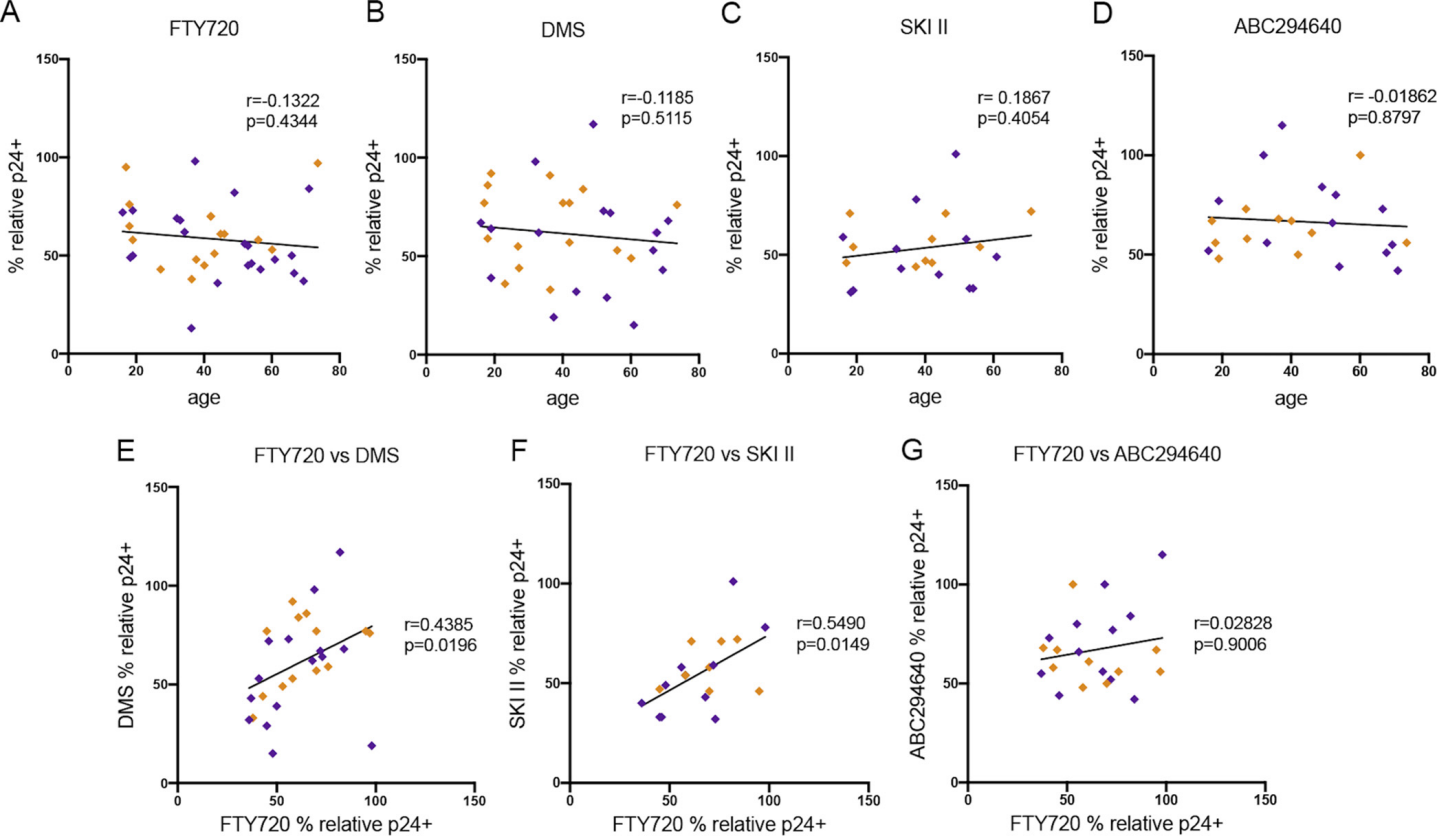
Effect of donor age on sphingosine kinase inhibition. (A) Correlation analysis of the relative p24^+^ population with 100 nM FTY720 treatment for 72 h during crowding versus donor age, *n* = 37 (*r* = −0.1322 and *P* = 0.4344). (B) Correlation analysis of the relative p24^+^ population with 3 μM DMS treatment for 72 h during crowding versus donor age, *n* = 33 (*r* = −0.1185 and *P* = 0.5115). (C) Correlation analysis of the relative p24^+^ population with 10 μM SKI II treatment for 72 h during crowding versus donor age, *n* = 22 (*r* = 0.1867 and *P* = 0.4054). (D) Correlation analysis of the relative p24^+^ population with 20 μM ABC294640 treatment for 72 h during crowding versus donor age, *n* = 24 (*r* = −0.01862 and *P* = 0.8797). (E) Correlation analysis of the relative (to untreated) p24^+^ population with FTY720 versus with DMS, *n* = 28 (*r* = 0.4385 and *P* = 0.0196). (F) Correlation analysis of the relative (to untreated) p24^+^ population with FTY720 versus with SKI II, *n* = 18 (*r* = 0.5490 and *P* = 0.0149). (G) Correlation analysis of the relative (to untreated) p24^+^ population with FTY720 versus with ABC294640, *n* = 22 (*r* = 0.02828 and *P* = 0.9006). For all correlation plots, male donors are denoted with purple symbols and female donors with golden brown.

We next investigated whether the age of the donors from which CD4 T cells were expanded contributed to the potency of the inhibitory effect of DMS, SKI II, ABC294640, or our control, FTY720, on HIV-1 cell-to-cell transmission. We have previously shown that age influences cell-to-cell transmission in CD4 T cells isolated from female donors but not male donors (32). As such, age could impact the effectiveness of HIV inhibitors. As described above, we generated CD4 T cells from donors of a range of ages, infected with NL4-3 and treated for 72 h while crowding with 3 μM DMS, 10 μM SKI II, 20 μM ABC294640, or 100 nM FTY720 (Fig. 1A, schematic). We then searched for a potential correlation between donor age and the relative percent p24^+^ population with SPHK inhibitor (or FTY720) treatment. As can be seen in Fig. 5A, there was no correlation between donor age and effect of FTY720 treatment on inhibition of HIV-1 infection in our sample population (age range, 19 to 75 years). Similarly, there was no correlation between DMS-mediated inhibition of infection and donor age (age range, 19 to 75 years) (Fig. 5B). SKI II (Fig. 5C) and ABC294640 (Fig. 5D) potency did not vary by donor age. Taken together, there was no correlation in our sample between donor age and the percent reduction of p24 with any of the inhibitors, echoing the equal effectiveness of SPHK/S1PR inhibition across biological sexes (Fig. 4). We next examined whether there are donor-specific differences in efficacy of the various pharmacologic modulators by searching for a potential correlation in the relative percent p24^+^ population following treatment with different inhibitors in the same donor. Interestingly, there was a significant correlation in the effectiveness of FTY720 and DMS as well as that of FTY720 and SKI II when considering the relationship between the effect of each treatment in the same donor (Fig. 5E and F), but there was no correlation observed between the effectiveness of FTY720 and ABC294640 (Fig. 5G), indicating potential donor-dependent factors independent of biological sex and age contributing to the response to inhibition of S1P receptors/SPHK and, as observed in Fig. 3, a potentially different mechanism of HIV inhibition of ABC294640.

### Pharmacologic inhibition and knockdown of SPHK1/2 reduce pSAMHD1 in CD4 T cells.

We have previously demonstrated that FTY720 reduces HIV-1 cell-to-cell transmission by multiple mechanisms, including by reducing CD4 surface density and altering the phosphorylation state of the innate restriction factor SAMHD1 in CD4 T cells, promoting an increase in unphosphorylated (active) SAMHD1. To investigate the mechanism by which S1P signaling inhibition reduces HIV-1 infection of primary CD4 T cells, we examined expression and surface density of CD4, CXCR4, and CCR5, as well as expression of the innate HIV-1 restriction factor SAMHD1, within uninfected CD4 T cells treated from days 10 to 13 with DMS, SKI II, ABC294640, or FTY720 (14). We optimized a flow cytometry stain for phosphorylated SAMHD1 (pSAMHD1; residue T592) in primary CD4 T cells and verified the level of pSAMHD1 at various time points of our model.

**FIG 6:**
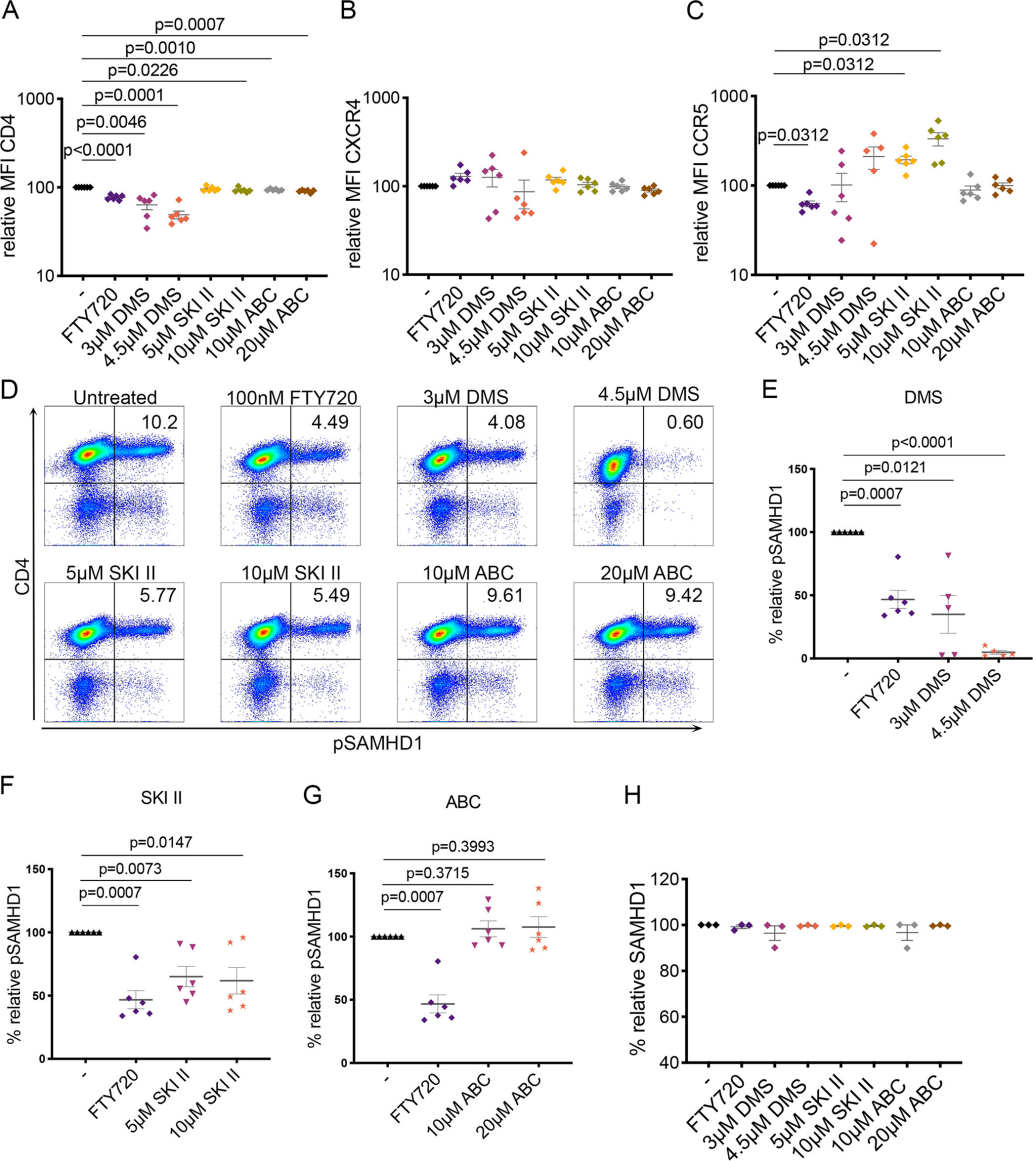
Sphingosine kinase inhibition reduces SAMHD1 phosphorylation in CD4 T cells. (A) Summary of relative MFI of CD4 in the CD4^+^ population following 72-h treatment with 100 nM FTY720, 3 or 4.5 μM DMS, 5 or 10 μM SKI II, and 10 or 20 μM ABC294640 (or no treatment), *n* = 6 (Wilcoxon matched-pair signed-rank tests for all comparisons). (B) Summary of relative MFI of CXCR4 in the CD4^+^ population following 72-h treatment with 100 nM FTY720, 3 or 4.5 μM DMS, 5 or 10 μM SKI II, and 10 or 20 μM ABC294640 (or no treatment), *n* = 6 (Wilcoxon matched-pair signed-rank tests for all comparisons). (C) Summary of relative MFI of CCR5 in the CD4^+^ population following 72-h treatment with 100 nM FTY720, 3 or 4.5 μM DMS, 5 or 10 μM SKI II, and 10 or 20 μM ABC294640 (or no treatment), *n* = 6 (Wilcoxon matched-pair signed-rank tests for all comparisons). (D) Representative flow cytometry analysis of phosphorylated SAMHD1 (pSAMHD1, residue T592) in uninfected CD4 T cells treated (or untreated) for 72 h with 100 nM FTY720, 3 or 4.5 μM DMS, 5 or 10 μM SKI II, and 10 or 20 μM ABC294640. (E) Summary of percent relative pSAMHD1 following 72 h treatment with 3 or 4.5 μM DMS and 100 nM FTY720, *n* = 6 (Wilcoxon matched-pair signed-rank tests). (F) Summary of percent relative pSAMHD1 following 72 h treatment with 5 or 10 μM SKI II and 100 nM FTY720, *n* = 6 (Wilcoxon matched-pair signed-rank tests). (G) Summary of percent relative pSAMHD1 following 72 h treatment with 10 or 20 μM ABC294640 and 100 nM FTY720, *n* = 6 (Wilcoxon matched-pair signed-rank tests). (H) Summary of relative percent total SAMHD1 following 72 h treatment with 100 nM FTY720, 3 or 4.5 μM DMS, 5 or 10 μM SKI II, and 10 or 20 μM ABC294640 (*n* = 3).

To examine CD4 and coreceptor expression, CD4 T cells were treated with 3 or 4.5 μM DMS, 5 or 10 μM SKI II, 10 or 20 μM ABC294640, or 100 nM FTY720 for 72 h and then harvested for flow cytometry analysis. Treatment with DMS significantly reduced the expression of CD4 (measured as relative mean fluorescence intensity [MFI]) on CD4 T cells, but there was not a striking reduction in CD4 MFI with SKI II or ABC294640 (Fig. 6A). Neither percent expression nor MFI of CXCR4 or CCR5 was significantly altered in the several donors examined, with the exception of SKI II treatment increasing CCR5 MFI (Fig. 6B and C).

We then examined the effect of SPHK modulation on phosphorylation of SAMHD1. CD4 T cells were treated with 3 or 4.5 μM DMS, 5 or 10 μM SKI II, 10 or 20 μM ABC294640, or 100 nM FTY720 for 72 h and then harvested for flow cytometry analysis of pSAMHD1 expression by phosphoflow staining. Following treatment with S1P signaling modulators, we observed that pSAMHD1 was significantly reduced with both 3 and 4.5 μM DMS and both 5 and 10 μM SKI II (relative phosphorylation of 34.92% and 4.95% for 3 and 4.5 μM DMS, 65.09% and 61.81% for 5 and 10 μM SKI II of that of the untreated control) as well as with FTY720 (46.77%) (Fig. 6D to F). However, ABC294640 treatment did not result in an appreciable alteration in pSAMHD1 expression (106.2% and 107.6% for 10 μM and 20 μM ABC294640) (Fig. 6D and G). The levels of total SAMHD1, also assessed by flow cytometry, remained consistent across all conditions (Fig. 6H).

**FIG 7:**
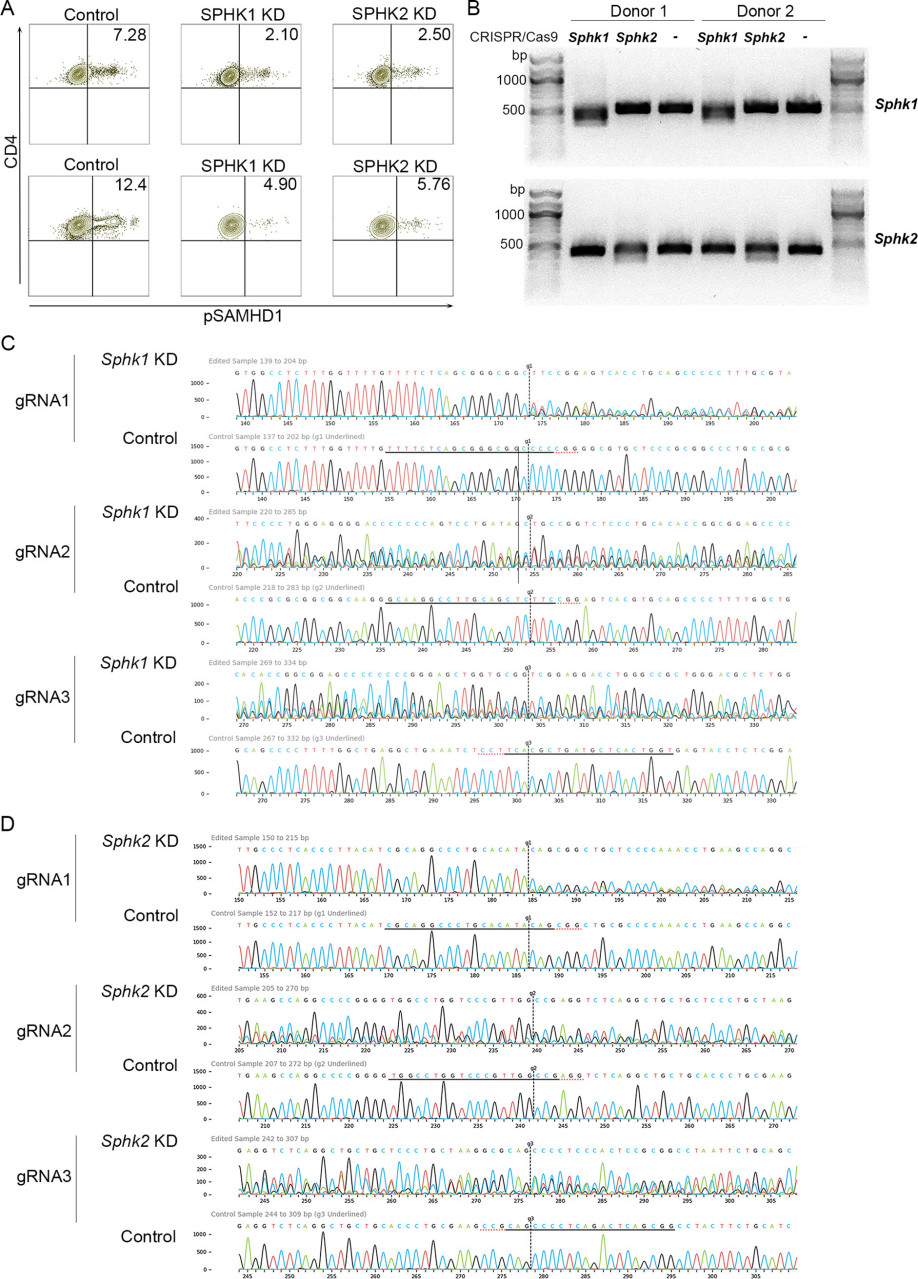
Sphingosine kinase knockdown reduces SAMHD1 phosphorylation in CD4 T cells. (A) Flow cytometry analysis of phosphorylated SAMHD1 (pSAMHD1, residue T592) in wild type/control (Cas9 only), SPHK1 knockdown (KD), and SPHK2 KD CD4 T cells (2 donors). (B) DNA gel electrophoresis demonstrating genetic disruption with *Sphk1-2* KD by CRISPR/Cas9 (1% agarose gel/TBE buffer). Top rows, *Sphk1* amplification; bottom rows, *Sphk2* amplification. (C) Knockdown analysis of *Sphk1* KD by CRISPR/Cas9 (Synthego) for one representative donor demonstrating sequence discordance between edited and control (wild type) samples. (D) Knockdown analysis of *Sphk2* KD by CRISPR/Cas9 (Synthego) for the same representative donor as panel C, demonstrating sequence discordance between edited and control (wild-type) samples. For both panels C and D, base calls from both edited and wild-type sample.ab1 files are shown. The black horizontal line represents the guide sequence and the red line denotes the PAM site, while the vertical black line shows the cut site. Mixed sequencing bases following the cut are normally observed due to cutting and error-prone repair. The analysis in panels C and D was performed in ICE software provided by Synthego.

To verify that SPHK targeting reduces pSAMHD1 in primary CD4 T cells, we performed multiguide sgRNA/Cas9-mediated knockdown of *Sphk1* or *Sphk2* in primary CD4 T cells by neon transfection electroporation. Forty-eight hours later, we quantitated pSAMHD1 (residue T592) by flow cytometry and observed reduced pSAMHD1 with both *Sphk1* and *Sphk2* knockdown compared to the Cas9 only control in two individual donors (Fig. 7A); this result was corroborated by single-pass Sanger sequencing demonstrating genetic disruption of *Sphk1* or *Sphk2* (Fig. 7B to D). In both donors, we observed successful knockdown of *Sphk1* (approximately 72% indels) and moderate knockdown of *Sphk2* (approximately 22 to 25%) relative to the wild type/Cas9 only control. These results indicate that both SPHK1 and SPHK2 regulate phosphorylation of SAMHD1 in CD4 T cells. Together, this suggests that DMS and SKI II reduce HIV transmission in CD4 T cells by lowering susceptibility to infection via reduction of SAMHD1 phosphorylation (resulting in a relative increase in the active form of the restriction factor), with a reduction in CD4 surface density by DMS contributing to the inhibitory effect of this compound. In contrast, the lack of alteration of pSAMHD1 via targeting SPHK2 with ABC294640 compared to the reduction observed with genome editing supports our data demonstrating that ABC294640 does not reduce the susceptibility of target cells to infection by a panel of HIV-1 primary isolates and molecular clones as FTY720, DMS, and SKI II do in pretreatment experiments (Fig. 3). In conclusion, we demonstrate that SPHK1 and SPHK2 regulate the levels of SAMHD1 phosphorylation in primary CD4 T cells and that pharmacologic targeting of SPKHs reduces the susceptibility of CD4 T cells to HIV infection.

## DISCUSSION

In this work, we examined whether targeting SPHK to reduce conversion of sphingosine to S1P could alter the course of HIV-1 infection in primary CD4 T cells. S1P binds to S1PR on CD4 T cells to initiate various signaling cascades (10), and we have previously demonstrated that inhibition of S1PR on primary CD4 T cells inhibits HIV-1 infection (14). We therefore hypothesized that limiting the availability of S1P itself would hinder HIV-1 infection in CD4 T cells.

Our results demonstrate that targeting sphingosine kinases inhibits cell-to-cell transmission of a range of HIV-1 primary isolates and molecular clones. SPHK1/2 inhibition with the dual SPHK inhibitor DMS, SPHK1 inhibition with SKI II, and SPHK2 inhibition with ABC294640 reduced HIV-1 transmission between CD4 T cells, while FTY720, which we have previously studied (14), reduced cell-to-cell transmission among CD4 T cells as well as between macrophages and autologous CD4 T cells in coculture transmission assays (Fig. 1 and 2). Interestingly, when CD4 T cells were pretreated with S1P modulators, labeled with Cell Trace dye, and cocultured with CD4 T cells infected with a panel of HIV-1 primary isolates and molecular clones, FTY720, DMS, and SKI II inhibited transmission of virus to target cells, whereas ABC294640 did not recapitulate this effect (Fig. 3). Three potential explanations for this result are that (i) SPHK1 is more highly expressed and/or more bioactive in CD4 T cells than SPHK2, (ii) inhibition of SPHK1, but not SPHK2, promotes changes to the biochemical environment that reduce permissiveness to HIV-1 infection, and/or (iii) SPHK2 is not effectively targeted by ABC29460 to reduce susceptibility to infection at concentrations of this compound that maintain viability in primary CD4 T cells. Our results indicate that the inhibitory effects observed with ABC294640 are unrelated to its ability to modulate SPHK2 at the concentrations tested and potentially due to off-target effects of the compound. Of note, the kinetics and metabolism of the inhibitors in primary CD4 T cells may differ in pretreatment versus continuous treatment, contributing to the different efficacy observed in the two contexts.

The SPHK isoenzymes, both present in CD4 T cells (33, 34), have distinct subcellular expression, with SPHK1 mainly present in the cytoplasm and SPHK2 in the nucleus. Subcellular localization appears to determine whether their contributions to S1P levels have pro- or antiapoptotic consequences, with Maceyka et al. finding that specific downregulation of SPHK1 increased S1P conversion to ceramide (decreasing S1P levels), whereas downregulation of SPHK2 had the opposite effect (17). As specific targeting of SPHK2 likely requires nuclear import of DMS/ABC294640, the efficiency with which this occurs in CD4 T cells may have implications for inhibition by the various compounds. Decreased conversion of sphingosine to S1P via SPHK inhibition supports our model of S1P promoting HIV-1 infection in CD4 T cells, which our previous work indicates (14). However, a detailed examination of protein-level expression as well as relative activity of the sphingosine kinases and kinetics of their inhibitors in CD4 T cells has yet to be performed, and this is an important future direction in determining the full mechanism of our observations.

The effect of SPHK inhibition on limiting HIV-1 infection in CD4 T cells was observed irrespective of the biological sex or age of the donor (Fig. 4 and 5), with neither the variable of biological sex nor that of age reducing the effectiveness of SPHK inhibition. Although sample size was somewhat limited after segregating into male and female donors, our specimens encompassed a wide range of ages within both biological sexes (males, 18.3 to 69.4 years of age; females, 17.9 to 73 years of age), and no correlation between age and effectiveness was observed with FTY720, DMS, SKI II, or ABC294640. Taken together, this indicates that inhibition of SPHK would be effective at reducing HIV-1 infection in donors of either sex or at any age, although additional exploration into factors influencing SPHK blockade are needed. Of note, there are also likely non-age- or sex-related donor-intrinsic factors, such as different expression levels of SPHK, S1P, or S1PR or sensitivity to/metabolism of S1P signaling modulators, that contribute to effectiveness of S1P receptor/SPHK modulation, as the effectiveness of FTY720 and DMS, as well as that of FTY720 and SKI II, were correlated when comparing the relative percent p24 following treatment in each donor (Fig. 5E to G).

**FIG 8:**
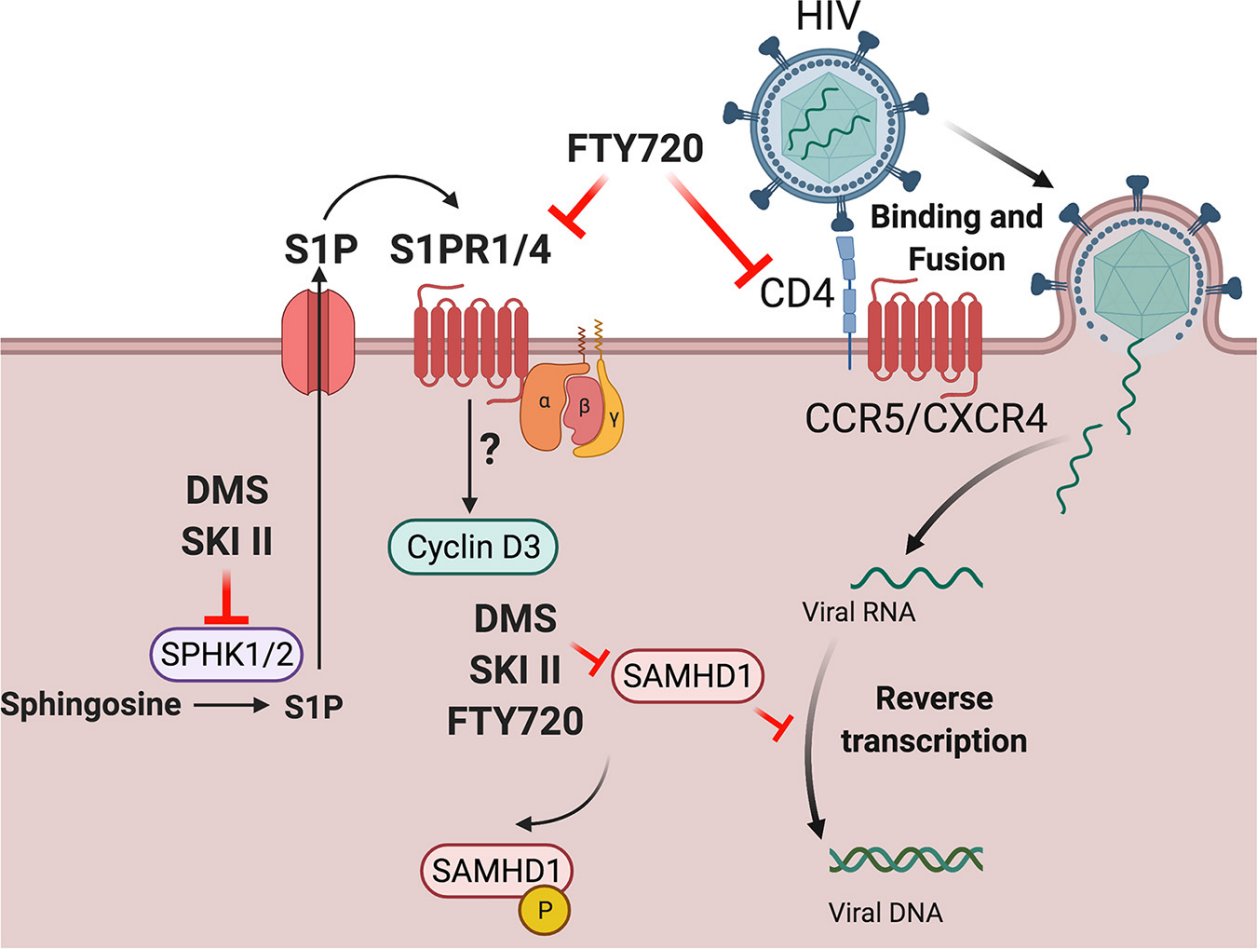
Summary of current model of the effect of inhibition of S1PR and SPHK in HIV-1 infection in CD4 T cells. Sphingosine kinases 1 and 2 (SPHK1/2) catalyze the phosphorylation of sphingosine to sphingosine-1-phosphate (S1P). These kinases are targeted by DMS (a dual inhibitor of SPHK1/2), SKI II (an inhibitor of SPHK1), and ABC294640, an inhibitor of SPHK2. FTY720 modulates expression of S1PR1, -3, -4, and -5, causing receptor internalization and precluding binding of S1P to its receptors. Our current model of SPHK inhibition in HIV-1 infection suggests that although DMS, SKI II, and ABC29460 all target SPHK1/2, only DMS and SKI II (along with FTY720) reduce HIV-1 infection in primary CD4 T cells by targeting SPHK, which appears to occur via a reduction in levels of phosphorylated (inactive) SAMHD1. This increased SAMHD1 activity is likely the mechanism for the reduction of HIV-1 infection with SPHK inhibitor pretreatment in coculture/pretreatment models as well as during cell-to-cell transmission. This image was created with Biorender.com.

SPHK-mediated inhibition appears to reduce susceptibility to HIV-1 infection mainly by downregulating phosphorylation of SAMHD1, the inactive state of the innate HIV-1 restriction factor. We have previously demonstrated by protein analysis that FTY720 reduces pSAMHD1 while maintaining total SAMHD1 levels (14), supporting the validity of our flow cytometry-based analysis, as we observed the same effect with our FTY720 control here. DMS, SKI II, and FTY720 all reduced relative pSAMHD1 expression. However, ABC294640 treatment did not reduce relative pSAMHD1 expression. Total SAMHD1 expression remained, for the most part, consistent with all treatments (Fig. 6H), indicating that SPHKI inhibition with DMS and SKI II (as well as S1PR inhibition with FTY720) increases the relative expression of the active form of the restriction factor. These results are in agreement with the reduction of HIV-1 p24-Gag observed with DMS, SKI II, and FTY720 pretreatment but lack of reduction of intracellular p24 with ABC294640 pretreatment in labeling/coculture experiments (Fig. 3), further supporting the proposed mechanism of HIV-1 infection inhibition occurring via a relative increase in SAMHD1 activation targeting preintegration events in the HIV-1 life cycle and reducing susceptibility to infection. This model is expanded by our demonstration that both SPHK1 and SPHK2 knockdown via genome editing reduced pSAMHD1 (Fig. 7), in contrast to the lack of pSAMHD1 alteration by ABC294640. Together, our data suggest that pharmacologic modulation of SPHK, especially SPHK1, is a feasible tactic for blocking HIV-1. Our observations and working model of the role of SPHK in HIV-1 infection are summarized in Fig. 8.

Taken together, and in light of our previous report that S1PR inhibition reduces HIV-1 infection, our results indicate that the conversion of sphingosine to S1P by SPHK facilitates HIV-1 infection of CD4 T cells. Our research suggests that therapeutic targeting of S1P availability early in infection via SPHK inhibition and phosphorylated SAMHD1 modulation will aid in the development of strategies to prevent establishment of initial infection and hinder cell-to-cell transmission of HIV-1 in CD4 T cells.

## MATERIALS AND METHODS

### Ethics statement.

Cells were isolated from buffy coats of anonymous healthy blood donors obtained from the Gulf Coast Regional Blood Center (GCRBC) in Houston, Texas. GCRBC provided us with only the biological sex and age of donors. Donors were at least 17 years old at the time of donation. No other personal identifiable information (including race/ethnicity) was provided.

### Reagents.

The following reagents were obtained through the AIDS Research and Reference Reagent Program, Division of AIDS, National Institute of Allergy and Infectious Diseases (NIAID), NIH: HIV-1 AD8 infectious molecular clone [pNL(AD8)], ARP-11346, contributed by Eric O. Freed; HIV-1, strain NL4-3 infectious molecular clone (pNL4-3), ARP-2852, contributed by M. Martin; HIV-1, strain JR-CSF infectious molecular clone (pYK-JRCSF), ARP-2708, contributed by Irvin S. Y. Chen and Yoshio Koyanagi; HIV-1, Z3678F infectious molecular clone (SGA 11), ARP-13269, contributed by Eric Hunter; HIV-1, 56313 (98US_MSC5016) virus, ARP-11253, contributed by Victoria Polonis; and HIV-1, 89/SO/145 (GS 016) virus, ARP-7697, contributed by Nelson Michael. The following HIV-1 primary isolates were a gift from Beatrice H. Hahn: p9004SDM, p190049, and p191882.

Human α-interleukin-12 (αIL-12) and αIL-4 were purchased through PeproTech. Human recombinant IL-2 (rIL-2) was obtained through the BRB/NCI Preclinical Repository. Antibodies were purchased from BD (CD4-allophycocyanin [APC]), Beckman Coulter (Kc57-fluorescein isothiocyanate [FITC]), BioLegend (CCR5-BV786), Cell Signaling (pSAMHD1), Origene (SAMHD1), and eBiosciences (eF450 fixable viability dye, CXCR4-phycoerythrin [PE]-Cy7). Cell Trace Yellow was purchased from ThermoFisher (number C34567). FTY720 (fingolimod/gilenya), DMS, SKI II, and ABC294640 were obtained from Cayman Chemical (FTY720, number 10006292, CAS number 162359-56-0; DMS, number 62575, CAS number 119567-63-4; SKI II, number 10009222, CAS number 312636-16-1; ABC294640, number 10587, CAS number 915385-81-8).

Cas9 nuclease/sgRNA gene knockout kits (V2) against *Sphk1* and *Sphk2* were purchased from Synthego (CA).

### Cell culture, generation of primary cell model of HIV-1, CRISPR/Cas9 knockdown, and inhibitor treatment.

Human peripheral blood mononuclear cells were obtained from healthy, HIV-negative, unidentified blood donors (GCRBC). Naive CD4 T cells were isolated from PBMCs by negative selection and activated in nonpolarizing conditions at 0.5 × 10^6^ cells/mL in the presence of 2 μg/mL α-human IL-12, 1 μg/mL α-human IL-4, 10 ng/mL transforming growth factor beta (TGF-β), and αCD3/28 stimulation beads at one bead/cell (Dynal/Invitrogen, CA) as previously performed (14, 27, 35). Subsequently, cells were expanded with 30 IU/mL human IL-2 in RPMI supplemented with 1% l-glutamine, 10% fetal bovine serum, and 1% penicillin-streptomycin.

In continuous treatment/cell-to-cell transmission experiments, incubation with SPHK inhibitors or FTY720 was performed on infected cells for 24 to 72 h (days 10 to 13) at concentrations of 100 nM to 20 μM, depending on the inhibitor. In Cell Trace/pretreatment experiments, incubation with SPHK inhibitors or FTY720 was performed on uninfected cells for 48 h (days 10 to 12) at concentrations of 100 nM to 20 μM, depending on the inhibitor, and treatment was removed prior to labeling with Cell Trace and then immediately coculturing with precrowded, infected CD4 T cells for 48 h (days 12 to 14).

CRISPR/Cas9 gene editing of primary CD4 T cells was performed by neon transfection electroporation of Synthego Cas9/sgRNA ribonucleoproteins (RNPs) according to manufacturer suggestions.

Macrophages were derived from PBMC donors in the presence of 100 ng/mL M-CSF and 10% sodium pyruvate (modification of references 30 and 31) and were cocultured with CD4 T cells pretreated with 100 to 200 nM FTY720 as described above.

### Generation of viruses.

For the HIV-1 infectious molecular clones pNL4-3, pJRCSF, pNLAD8, pZ3678F, p9004SDM, p190049, and p191882, infectious virus was generated in HEK293FT cells by calcium phosphate transfection as described previously (27, 35). The primary isolates HIV-1 90/SN/364 (ARP-7696) and HIV-1 56313 (ARP-11253) were expanded in phytohemagglutinin (PHA)-activated PBMCs as described above. Following rescue in PHA, activated PBMCs were cultured for 3 to 4 days with 2 μg/mL Polybrene, 15% fetal bovine serum, 20 IU IL-2/mL, 2 mM l-glutamine, and 1% streptomycin and penicillin, resuspended with virus stock for 30 min at 37°C, and then resuspended in fresh medium for 3 to 4 days. Twice weekly, cultures were fed with either fresh PBMCs or coculture medium for up to 35 days (36).

### Flow cytometry.

To analyze productively infected cells, 2.5 × 10^5^ cells were stained for CD4 (clone S3.5, APC; BD), fixable viability dye (eF450; eBiosciences), and intracellular p24-Gag (Kc57, FITC; BD) and were analyzed on a Celesta flow cytometer (BD) as previously performed, with 1.5 × 10^5^ events collected per condition (35). To label cells prior to coculture assays, cells were stained with Cell Trace Yellow proliferation dye (ThermoFisher, MA) according to the manufacturer’s instructions. To determine percent expression of total and phosphorylated SAMHD1, CD4 T cells were stained with pSAMHD1 (Cell Signaling Technologies) and total SAMHD1 (Origene) according to manufacturer protocols. For determination of CD4 and coreceptor MFI, cells were stained for CD4 (clone S3.5, APC; BD), fixable viability dye (eF450; eBiosciences), CXCR4 (PE-Cy7; eBiosciences), and CCR5 (BV786; BioLegend) and were analyzed on a Celesta flow cytometer (BD) as previously performed (35).

### Sequencing and analysis.

Flow cytometry analysis was performed in FlowJo software (BD). All statistical analyses were performed in GraphPad Prism. Single-pass Sanger sequencing was performed with ACGT sequencing on gel-purified PCR products following neon electroporation of sgRNA/Cas9 RNPs and *Sphk1-2* amplification, and analysis of indels/sequence disruption was performed with ICE software, provided by Synthego.
